# *WT1* complete gonadal dysgenesis with membranoproliferative glomerulonephritis: case series and literature review

**DOI:** 10.1007/s00467-022-05421-8

**Published:** 2022-02-24

**Authors:** Erin Anderson, Melanie Aldridge, Ross Turner, James Harraway, Sam McManus, Anna Stewart, Peter Borzi, Peter Trnka, John Burke, David Coman

**Affiliations:** 1 Queensland Fertility Group, Virtus Genetics, Brisbane, Australia; 2 Department of Nephrology, The Queensland Children’s Hospital, Brisbane, Australia; 3 Monash IVF, The Wesley Hospital, Brisbane, Australia; 4 Mater Pathology, The Mater Hospital, Brisbane, Australia; 5 Department of Anatomical Pathology, The Royal Brisbane and Women’s Hospital, Brisbane, Australia; 6 Department of Paediatric Surgery and Urology, The Queensland Children’s Hospital, Brisbane, Australia; 7 Department of Paediatrics, The Wesley Hospital, Brisbane, Australia; 8 The School of Medicine, The University of Queensland, Brisbane, Australia; 9 Department of Metabolic Medicine, The Queensland Children’s Hospital, 501 Stanley Street, South Brisbane, QLD 4101 Australia; 10 The School of Medicine, Griffith University, Gold Coast, Australia

**Keywords:** WT1, Membranoproliferative glomerulonephritis (MPGN), Frasier syndrome, Disorders of sex development

## Abstract

**Background:**

Intronic *WT1* mutations are usually causative of Frasier syndrome with focal segmental glomerulosclerosis as the characteristic nephropathy. Membranoproliferative glomerulonephritis is not commonly associated with disorders of sex development but has been recently identified as a *WT1*-associated nephropathy, but usually in cases of exonic mutations in either isolated Wilms tumor or Denys-Drash syndrome.

**Methods:**

The clinical and genetic data from 3 individuals are reported.

**Results:**

This report describes the kidney manifestations in 3 individuals from 2 unrelated families with Frasier syndrome intronic *WT1* mutations, noting that 2 of the 3 individuals have histologically confirmed membranoproliferative glomerulonephritis.

**Conclusions:**

These case reports support expansion of the clinical spectrum of the kidney phenotypes associated with Frasier syndrome providing evidence of an association between *WT1* mutation and an immune complex-related membranoproliferative glomerulonephritis.

**Graphical abstract:**

A higher resolution version of the Graphical abstract is available as Supplementary information

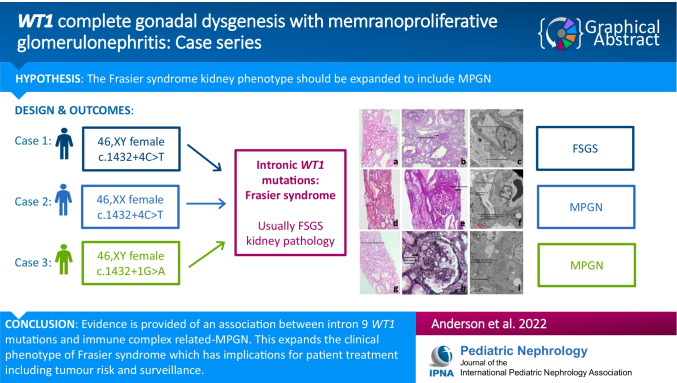

**Supplementary Information:**

The online version contains supplementary material available at 10.1007/s00467-022-05421-8.

## Introduction

The Wilms tumor suppressor gene (*WT1*, OMIM 607,102) encodes a zinc finger transcription factor involved in early kidney and urogenital development, which is inactivated in patients with pediatric kidney cancer. Functional WT1 protein is required for embryonic gonadal development. It is highly expressed in the fetal kidney and is required for the development of the glomerulus. Ongoing expression is required in mesothelial and podocyte cells for functional regulation including cell differentiation, function, and structural integrity. Mutations involving *WT1* have been associated with various glomerulopathies including focal segmental glomerulosclerosis (FSGS) and diffuse mesangial sclerosis (DMS) [1].

*WT1*-related nephropathy can occur in isolation, or as part of urogenital malformation syndromes and disorders of sex development, including Wilms tumor, aniridia, genitourinary anomalies, mental retardation syndrome (WAGR, OMIM 194,072); Frasier syndrome (FS, OMIM 136,680); Denys-Drash syndrome (DDS, OMIM 194,080); and, rarely, Meacham syndrome (MS, OMIM 608,978), the latter 3 being allelic disorders with an overlap of clinical features [1]. FS is characterized by gonadal dysgenesis, development of FSGS nephropathy, progressive chronic kidney disease (CKD) often leading to kidney failure, and a high risk of gonadoblastoma. DDS also presents with gonadal dysgenesis but has an earlier onset and more rapid progression of DMS nephropathy, nephrotic syndrome, and Wilms tumor risk.

*WT1* genotype–phenotype correlations exist with whole-gene deletions at 11p13 associated with WAGR [2], intron 9 splice site point mutations associated with FS [3], exon 8 or 9 zinc finger dominant-negative missense mutations associated with DDS [4], and nonsense mutations causing partial kidney or gonadal developmental defects such as isolated Wilms tumor or nephrotic syndrome [5].

Gonadal dysgenesis is a hallmark of both FS and DDS, but these syndromes are not typically considered in an initial differential diagnosis when investigating childhood membranoproliferative glomerulonephritis (MPGN). This type of kidney pathology usually occurs secondary to systemic immune disorders, chronic infection, or cancer [6] and, in lieu of a clear etiology, often results in a diagnosis of idiopathic nephrotic syndrome. MPGN is a common cause of CKD and can present with both nephritic and/or nephrotic phenotypes. It is associated with immune complex deposition injury of the glomerulus and chronic complement activation [6]. These immune deposits in the glomerular mesangium and capillary walls cause injury with capillary thickening and mesangium enlargement, characteristic of MPGN [7] with subsequent development of nephrotic syndrome. Chronic complement activation can also lead to kidney failure through acute hemolytic uremic syndrome (aHUS) due to platelet activation, endothelial cell damage, and white blood cell activation. In addition, aHUS can be associated with *WT1* mutations, in particular with exon 9 zinc finger mutations as a manifestation of DDS [8–10].

Herein, we report 3 patients from 2 families with FS due to characteristic intron 9 *WT1* mutations and unusual nephropathy of MPGN.

## Case reports

### Cases 1 and 2

#### Case 1

**Fig. 1:**
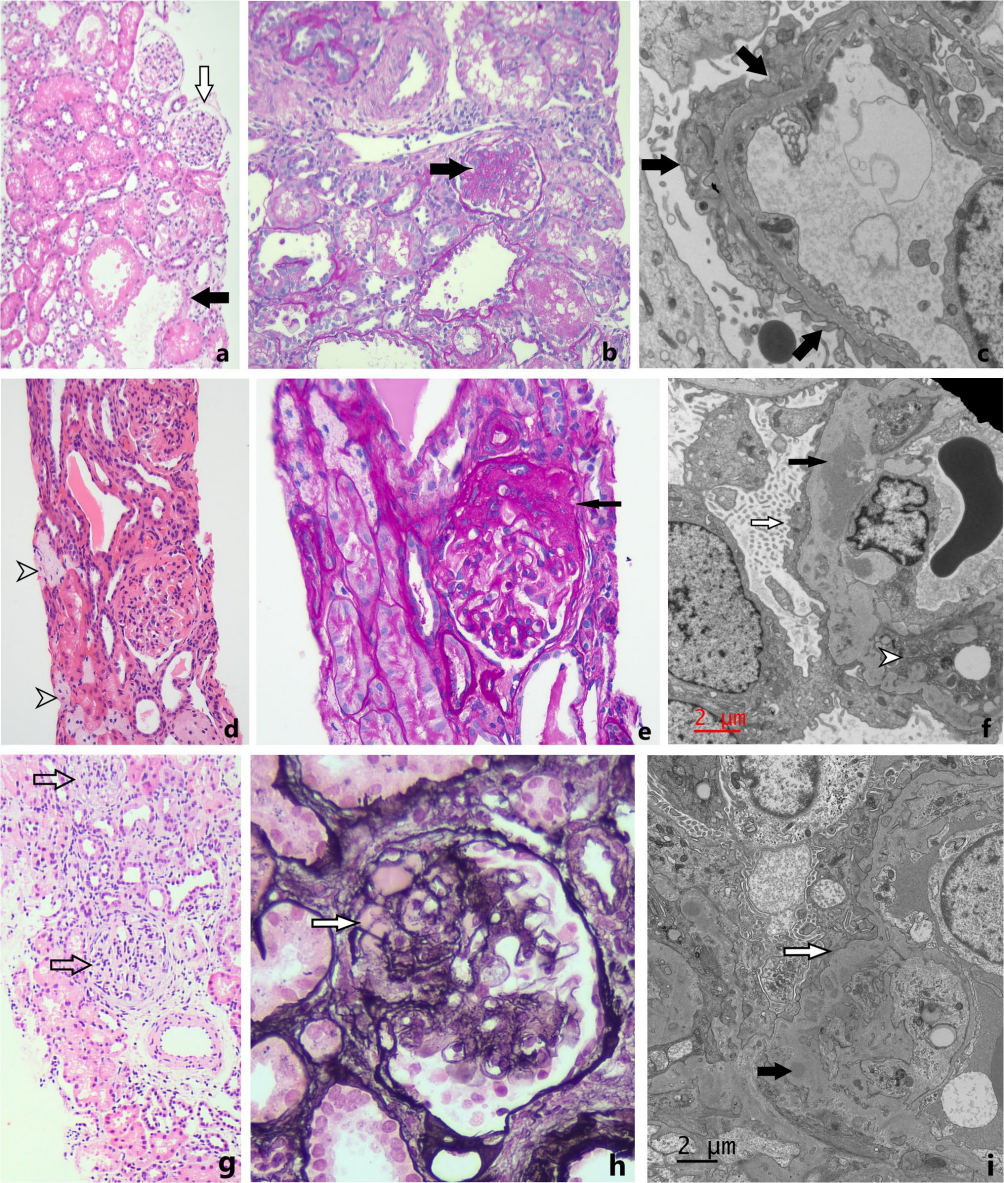
Light and electron microscopy histological findings of kidney biopsy samples obtained from the cases with *WT1* intronic mutation and membranoproliferative glomerulonephritis. Case 1: **a** Renal cortex including normal glomeruli (white arrow) and dilated tubules (black arrow). **b** Single glomerulus showing focal segmental sclerosis (arrow). **c** Electron micrograph showing abnormal basement membrane (arrows). Case 2: **d**. Foamy macrophages (arrowheads). **e**. Segmental sclerosis within the glomerulus highlighted by PAS stain (arrow).** f**. Electron micrograph showing electron dense deposits (black arrow), foot process effacement (white arrow), and segmental interposition of cells within reduplicated glomerular basement membrane (white arrowhead). Case 3: **g**. Renal cortex showing glomeruli with mesangial hypercellularity (arrows). **h**. Segmental sclerosis with hyalinosis highlighted by silver stain (arrow). **i**. Electron micrograph showing foot process effacement with microvillous transformation (white arrow) and electron dense deposit (black arrow)

The proband was initially brought to attention after a noninvasive prenatal testing predicted a 46,XY karyotype with a female phenotype subsequently observed on tertiary-level ultrasound. Amniocentesis confirmed the 46,XY karyotype and identified a heterozygous pathogenic c.1432 + 4C > T variant in the intron 9 splice site region of the *WT1* gene. The infant was delivered via spontaneous vaginal delivery at term and was phenotypically female indicating 46,XY complete gonadal dysgenesis. Abdominal and pelvic ultrasound demonstrated a normal uterus and did not visualize any gonadal tissue. By 17 weeks of age, the baby already had significant proteinuria of 450 mg/L (ref. < 100 mg/L) and a protein-creatinine ratio (UPCR) of 499 mg/mmol creatinine (ref. < 15 mg/mmol). Kidney function was normal with serum creatinine (< 30 mmol/L) and albumin (35 g/L). At 11 months of age, urine protein concentration increased to 2000 mg/L with a UPCR of 1954 mg/mmol. Investigative laparoscopy at 6 months of age identified streak gonads which were removed due to the risk of gonadoblastoma. Histopathology of gonads showed dense ovarian cortex-like stroma with epithelial cord-like structures, admixed germ cells and sex cord-stromal cells. No ovarian follicles were identified and clusters of probable Leydig cells were seen in the hilar region. A kidney biopsy was performed at 14 months of age. Light microscopy demonstrated FSGS with 1 of 11 glomeruli showing segmental sclerosis (in keeping with focal segmental sclerosis), and focal proximal tubular hypertrophy with cystic dilatation of the tubules. Immunofluorescence was negative. Immunohistochemistry showed normal staining for WT1 in podocytes. Electron microscopy showed an abnormal glomerular basement membrane with irregular basket weave-like lamination and irregular thinning (Fig. 1a–c).

#### Case 2

The biological mother of case 1 presented at 12 years of age with steroid-resistant nephrotic syndrome (SRNS) following an intercurrent illness. Investigations demonstrated normal kidney function, normal complement, and normal serum albumin. Serology for systemic lupus erythematosus and ANCA vasculitis was negative, but she had microscopic hematuria and significant proteinuria (7.5 g protein in 24-h urine collection). Kidney biopsy was performed. The light microscopy of 16 glomeruli per section showed global sclerosis of 3 glomeruli, and segmental sclerosis of 4 glomeruli and focal hyalinosis. Immunofluorescence demonstrated diffuse granular glomerular capillary wall IgA, IgM, and C3 deposits. Electron microscopy showed segmental subendothelial deposits associated with mesangial interposition, markedly thickened capillary walls, extensive effacement of foot processes, and villous transformation of epithelial cells. She was diagnosed with idiopathic type 1 MPGN. Treatment with 4 weeks of prednisolone and 6 months of ciclosporin was ineffective; she progressed to kidney failure and eventually received a kidney transplant. By the time of planning for this pregnancy, the transplant function was declining and a surrogate was utilized to carry her biological embryo (case 1). In view of the antenatal diagnosis of a disorder of sex development syndrome of the fetus, cascade testing in the biological mother confirmed the same *WT1* mutation with a 46,XX karyotype.

#### Case 3

The phenotypically normal girl presented at age 5.5 years with nephrotic syndrome manifesting predominantly with ascites. Investigations showed normal kidney function, normal complement levels, and normal lupus serology and hypoalbuminemia (17 g/L [reference 35–55 g/L]). She had nephrotic range proteinuria but did not respond to treatment with prednisolone. A kidney biopsy was performed for ongoing nephrotic range proteinuria. The light microscopy showed extensive glomerular mesangial hypercellularity and mesangial matrix expansion, with at least 1 glomerulus showing segmental sclerosis with hyalinosis. Fifteen glomeruli were present in the biopsy (2 of which were globally sclerosed, and 3 appeared normal). The immunoflorescence was reported as IgG pseudo-linear peripheral capillary wall staining (2 +), IgM mesangial and some peripheral capillary wall reactivity (2 +) IgA same IgG, C3-negative, and C1q mesangial reactivity (1 +). Electron microscopy showed numerous subendothelial electron-dense deposits with cellular interpositions, glomerular basement membrane duplication, and foot process effacement with microvillus transformation, consistent with type 1 MPGN with immune complex deposits, no crescents and no C3 deposition. She continued to have significant proteinuria despite treatment with ciclosporin and ACE inhibitor, with ongoing decline in kidney function. The repeat biopsy shows persisting membranoproliferative pattern of injury in the glomeruli (15 glomeruli sampled) and developing chronic kidney disease with the chronic change present scored as 3/8. C apillary wall staining for IgG, IgA, IgM, C3, C1q.

Despite ongoing immunosuppression with ciclosporin and added mycophenolate, the girl progressed to kidney failure and eventually received a kidney transplant. Additional significant medical history for case 3 is that she developed bronchiectasis detected in the context of a chronic productive cough before she progressed to kidney failure. Investigations included a G-banded karyotype which unexpectedly demonstrated 46,XY. A pelvic ultrasound confirmed gonadal dysgenesis with presence of a uterus and bilateral gonad biopsies showed bilateral gonadoblastoma, with both gonads subsequently removed. Genetic testing identified a heterozygous c.1432 + 1G > A *WT1* variant.

## Materials and methods

Genetic testing for cases 1 and 3 was performed by Mater Pathology via massive parallel sequencing (MPS) as part of a disorder of sex development gene panel which included *WT1* (transcript LRG 525t1). Targeted enrichment was performed using Illumina-Nextera rapid capture probes, followed by MPS on an Illumina MiSeq and data analysis with CLC Genomics Workbench and Cartagenia Benchlab NGS. Confirmation of the pathogenic variant was performed for cases 1, 2, and 3 using Sanger sequencing via standard methods (Mater Pathology).

## Discussion

The effects of various *WT1* mutations on gonadal development, and on the development and ongoing function of the kidney, are due to its role as a transcriptional regulator for sex-determining genes such as *Sry* [11], and on podocyte-specific target genes such the nephrin-encoding *NPHS1* [12]. The DNA-binding zinc finger domains of the WT1 protein are encoded by exons 7–10 and mutations, particularly in exon 9, reducing DNA-binding affinity with subsequent dominant-negative functional inhibition [13] or aberrant WT1 protein dosage [14]. *WT1* deletion mutations have been linked to glomerulosclerosis through the Notch [15] and Wnt [16] signaling pathways.

The *WT1* intron 9 splice site contributes 2 of the 24 different WT1 protein isoforms [17, 18]. These 2 major splice isoforms are + KTS and − KTS with insertion or exclusion respectively of a lysine-threonine-serine sequence between the 2 terminal zinc finger DNA/RNA binding motifs. These 2 isoforms have differing nuclear roles, and maintenance of the finely balanced + KTS/ − KTS ratio is required for correct *WT1* function [3, 19] in both kidney and gonadal development. *WT1* modifies transcription of up to half of podocyte-specific genes, and correct KTS ratio balance is required for this [20]. As part of its role in *Sry*-based sex determination, the + KTS variant has different outcomes depending on karyotype. On a 46,XX background, a reduction in this isoform leads to isolated nephrotic syndrome only [21], whereas 46,XY individuals display complete gonadal dysgenesis and kidney pathology of FSGS [21]. The two 46,XY individuals presented in this report (cases 1 and 3) both demonstrate complete gonadal dysgenesis.

The intron 9 splice site mutations of *WT1* are associated with a high risk of gonadoblastoma and only rarely with Wilms tumor [21]. As well as FS, this particular *WT1* intron 9 splice site mutation has been atypically associated with DDS [22] and, conversely, there have been occasional patients diagnosed with FS with an atypical exonic *WT1* mutation [23]. These idiosyncratic cases highlight the lack of clear correlation between *WT1* mutations and urogenital pathology, and intronic mutations have been reported with FS [3, 19], DDS, and isolated DMS [24] and various atypical presentations including FS with Wilms tumor [25] and 46,XX females with FSGS only [22, 26].

The distinct familial nephropathies of cases 1 and 2 highlight the role of other genetic and environmental factors in phenotypic outcome. This has been similarly highlighted in a previously described inherited intron 9 *WT1* splice site mutation resulting in different glomerulopathies in a 46,XX mother (FSGS) and her 46,XY daughter (DMS) [22]. The diagnosis of isolated childhood nephrotic syndrome in case 2 was apt at the time given her normal pubertal development and the histological diagnosis of MPGN that was not specifically indicative of a genetic cause of nephrotic syndrome. Several cases are documented of MPGN upon initial kidney biopsy followed by FSGS on follow-up several years later [27, 28]. This highlights the complexity of histological diagnosis for Frasier syndrome and other nephropathies, as well as the dynamic relationship of genetic and environmental factors.

The screening for genetic variants in genes associated with nephrotic syndrome, including *WT1*, should be an integral part of investigations of nephrotic syndromes. The phenotypic and genotypic heterogeneity of *WT1*-associated disease presents challenges for clinicians, with patient care impacted due to the very different phenotypic continua, tumor risks, and surveillance programs required for FS and other nephropathies. Treatment options for SRNS are often limited but a clear molecular diagnosis provides avenues for best clinical support and patient outcome. The 3 cases presented here support broadening the diagnosis of FS to include genotypic females and MPGN nephropathy.

## Supplementary Information

Below is the link to the electronic supplementary material.

Graphical Abstract 5421 (PPTX 619 KB)

## Data Availability

Available upon request from the corresponding author.
