# Supplementary material for: WT1 complete gonadal dysgenesis with membranoproliferative glomerulonephritis: case series and literature review
Source: Pediatr Nephrol. 2022 Feb 24;37(10):2369–74. doi: 10.1007/s00467-022-05421-8 (PMC9395477; doi:10.1007/s00467-022-05421-8)
Supplement: Supplementary file 1 — Graphical Abstract 5421 (PPTX 619 KB) [file 467_2022_5421_MOESM1_ESM.pptx]

## Slide 1
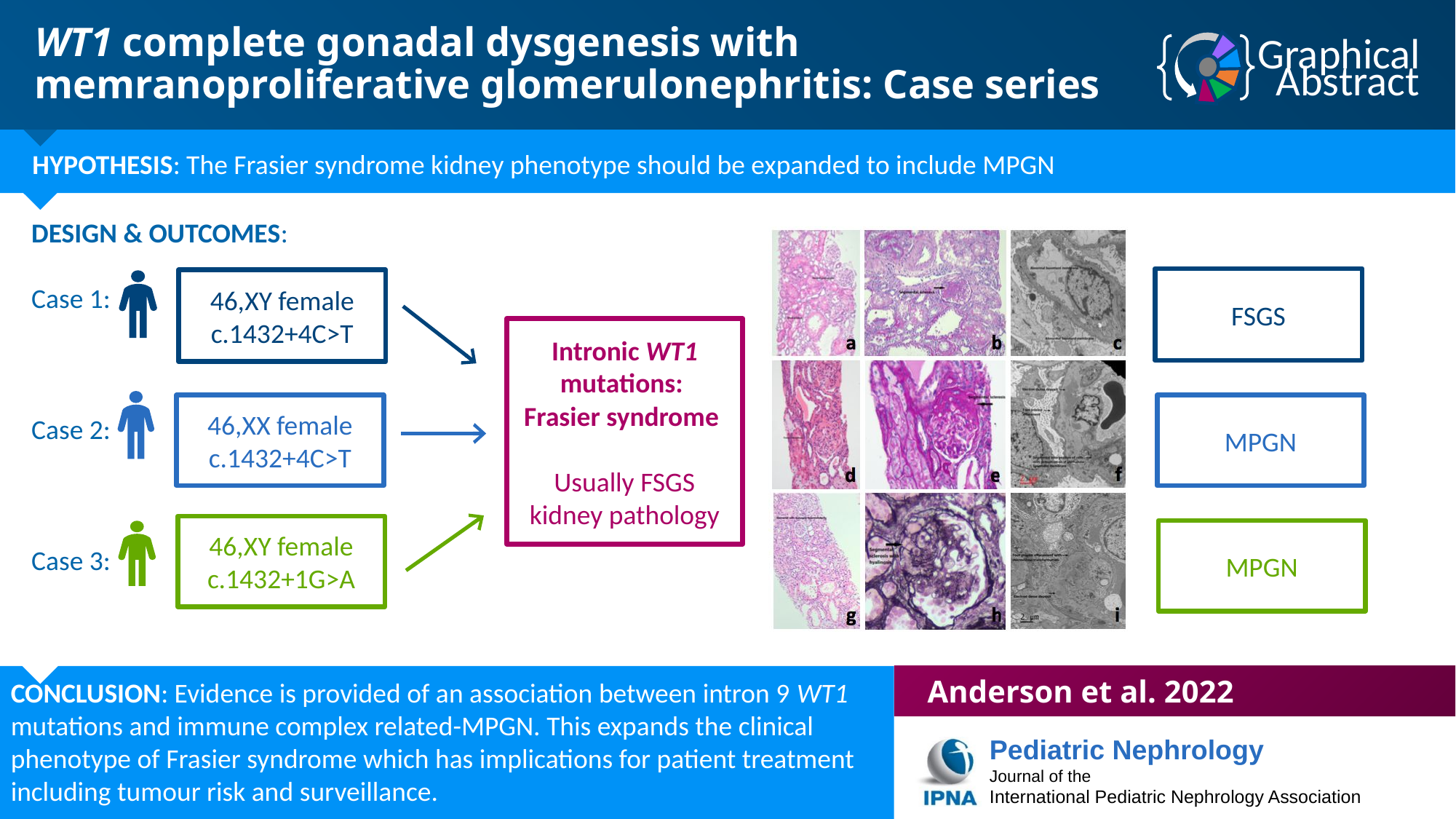

WT1 complete gonadal dysgenesis with memranoproliferative glomerulonephritis: Case series
HYPOTHESIS: The Frasier syndrome kidney phenotype should be expanded to include MPGN
DESIGN & OUTCOMES:
Case 1:
Case 2:
Case 3:
FSGS
46,XY female
c.1432+4C>T
Intronic WT1 mutations:
Frasier syndrome
Usually FSGS kidney pathology
46,XX female
c.1432+4C>T
MPGN
46,XY female
c.1432+1G>A
MPGN
Anderson et al. 2022
CONCLUSION: Evidence is provided of an association between intron 9 WT1 mutations and immune complex related-MPGN. This expands the clinical phenotype of Frasier syndrome which has implications for patient treatment including tumour risk and surveillance.
